# Pullulan-Based Spray-Dried Mucoadhesive Microparticles for Sustained Oromucosal Drug Delivery

**DOI:** 10.3390/pharmaceutics16040460

**Published:** 2024-03-26

**Authors:** Ting Liu, Xiang Gong, Yang Cai, Hao-Ying Li, Ben Forbes

**Affiliations:** 1 College of Stomatology, Guizhou Medical University, Guiyang 550004, China; liuting628@gmc.edu.cn (T.L.); caiyang85@gmc.edu.cn (Y.C.); 2 Guiyang Hospital of Stomatology, Guiyang 550007, China; gongxoralmed@hotmail.com; 3 Institute of Pharmaceutical Science, King’s College London, London SE1 9NH, UK

**Keywords:** pullulan, spray-dried microparticles, sustained drug release, mucoadhesion

## Abstract

Mucoadhesive microparticles for oromucosal drug delivery offer several advantages, including intimate contact with the mucosa, delivery to less accessible regions, extended residence time, sustained drug release, reduced irritation, and improved patient compliance. In this study, pullulan was used to prepare mucoadhesive spray-dried microparticles for delivering benzydamine hydrochloride (BZH) to oral mucosa. The BZH-pullulan spray-dried microparticles had a mean size of <25 μm with an angle of repose values between 25.8–36.6°. Pullulan markedly extended drug-release time to >180 min, ~9 times greater than the duration (i.e., 20 min) reportedly achieved by chitosan. Kinetic analysis showed the drug-release rate was concentration dependent and jointly controlled by drug diffusion and polymer chain relaxation. Further, pullulan was mucoadhesive and was able to retain up to 78.8% w/w of microencapsulated gold nanoparticle probes at the mucosal membrane. These data strongly suggest that BZH-pullulan microparticles have great potential for oromucosal drug delivery, by providing elongated residence time in situ and sustained drug release for the treatment of local diseases.

## 1. Introduction

Local drug delivery is the preferred approach for the treatment of oral mucosal diseases such as aphthous stomatitis and mucosal ulcers, as it produces high drug concentrations in situ using low doses and circumvents systemic side effects [1]. However, the oral environment, particularly the constant flow of saliva fluid (up to 1.5 L per day) [2] and continuous swallowing actions (with the mean of 580 and up to 1008 times per day) [3,4], detrimentally reduce the drug residence time on oral mucosa which lessens therapeutic efficacy. To combat these clearance mechanisms, effective oromucosal drug delivery requires that the formulation should have: (i) strong adhesion to the oral mucosa to resist the flush of saliva, thereby elongating drug residence time on-site; and (ii) sustained drug release to generate continuous local treatment for oral diseases over an extended period [5,6].

To meet these requirements, a mucoadhesive drug-delivery system (MDDS) has been developed. The construction of the MDDS was designed to enhance therapeutic efficacy by adhering to the mucosal membrane and slowly liberating the drug molecules from the polymer matrix to generate a prolonged therapeutic action [7,8,9]. A variety of MDDS have been developed for oromucosal drug delivery, including tablet, film, and patch dosage forms [10,11,12,13]. However, limitations of these dosage forms include that they have a fixed contact area, limited drug loading capacity, and lack the physical dimensions and flexibility to access recessed disease target regions such as the gingivae, lingual margins, and periodontal pocket. Additionally, tablets, films, and patches can produce a sense of discomfort for patients, particularly when applied repeatedly for long-term treatment, and some patches have to be removed from oral cavity after treatment, which can cause additional inconvenience for patients [14,15,16]. To solve these problems, MDDS microparticles have been developed for oromucosal drug delivery, with the additional advantage that microparticles (<38 μm) have been shown to have the capability to stick to the mucosal membrane [17,18]. Mucoadhesive drug microparticles can be conveniently administered to the oral target regions using powder sprays to entirely cover the affected regions and blanket the less accessible disease pockets with adherent drug-carrying microparticles. The ability of microparticles to reduce local irritation and avoid the inconvenience and mouth feel of other dosage forms can also improve patient compliance [19]. In our previous study [20], mucoadhesive chitosan-based microparticles were created for oromucosal drug delivery. It was confirmed that the use of mucoadhesive excipient of chitosan in the formulations enhanced the adhesion to mucosal membrane and effectively controlled the liberation of the drug molecules from the chitosan matrix to provide a sustained drug release. However, the duration of drug release was still limited (i.e., 20 min) and needs to be further improved.

Pullulan is a microbial exopolysaccharide, commercially produced from a fermentation process on starch by a fungus of *Aureobasidium pullulans.* Pullulan has the formula of (C_6_H_10_O_5_)_n_ with molecular weight (M.W.) of 4.5 × 10^4^–6 × 10^5^ Daltons. In chemical structure, pullulan is a linear, non-branched chain primarily constructed by repeating maltotriose units connected by α-1,6 glycosidic linkage, inside which glucose subunits are linked by α-1,4-glycosidic bonds [21,22]. Pullulan is water soluble and has well-established biocompatibility, biodegradability, non-toxicity, non-immunogenicity, and non-carcinogenicity [23,24], all of which are desirable features for a pharmaceutical excipient used in drug-delivery systems [25]. Besides, pullulan has remarkable mucoadhesivity that can enhance the drug retention at mucosal surfaces and has also been utilized as a polymer matrix for controlling the release of loaded drugs [26,27]. Prajapati et al. [28] employed pullulan to prepare zolmitriptan-loaded thin films which were mucoadhesive when applied to the tongue surface and produced controlled drug-release profiles to provide effective treatment for the targeted disease [28]. Additionally, Sallam et al. [29] fabricated pullulan-based films for sublingual delivery of salbutamol sulphate (SS), which, in comparison with tablets, offered the advantages of avoidance of first-pass metabolism and reduction in the *T_max_* to produce quicker onset of action. However, to date, pullulan-based particulate systems have not been developed for local oromucosal drug-delivery applications.

Spray drying, a one-step process, has proved to be an effective approach for engineering microparticles of a large range of active pharmaceutical ingredients, and offers the capability to regulate physiochemical properties of spray-dried microparticles, e.g., particle size distribution, surface morphology, moisture content, density, and polymorphs [30,31]. Benzydamine hydrochloride (BZH) is a non-steroidal anti-inflammatory drug, which is generally utilized as a medicine for the relief of pain and inflammation [32,33]. BZH can also act as a resident anesthetic, initiating numbness and relieving pain at the site of application in the oral cavity [34]. Consequently, BZH is well employed for dealing with oral mucosal diseases such as ulcers, mucositis, and burning mouth syndrome, and it is most effective if applied directly to the affected areas [35].

In this paper, spray drying was utilized to prepare microparticles that were aimed to deliver drugs directly to oromucosal membranes for treatment of local diseases. The mucoadhesive pullulan was employed in the formulations to enhance the mucoadhesion of drug particles to oral mucosa and to control the drug release. A series of spray-dried microparticles with systematically-varied BZH:pullulan mass ratios were prepared and evaluated for their physiochemical properties, including morphology, particle size distribution, and flowability, as well as key performance attributes which were assessed by in vitro drug release and ex vivo mucoadhesion.

## 2. Materials and Methods

### 2.1. Materials

BZH was purchased from China Langchem Inc. (Shanghai, China). Pullulan (M.W. 200,000 Daltons) was purchased from Sangon Biotech. (Shanghai, China). Gold nanoparticles (GNPs, 50 nm in diameter, with a concentration of 3.5 × 10^10^ particles/mL) were purchased from the Sigma-Aldrich Corporation (St. Louis, MO, USA). Disodium hydrogen phosphate, potassium dihydrogen phosphate, sodium acetate, and acetic acid were acquired from Sinopharm Chemical Reagent Co., Ltd. (Shanghai, China). Phosphoric acid was bought from Guangdong Guanghua Chemical Factory Co. Ltd. (Guangzhou, China). Acetonitrile at HPLC grade was obtained from Spectrum Chemical Mfg. Corp. (Gardena, CA, USA).

### 2.2. Preparation of Spray-Dried Microparticles

The drug and pullulan were precisely weighed in a series of mass ratios. Pullulan at a mass of 1000 mg was thoroughly dissolved in 200 mL of deionized water to produce a solution of 5 mg/mL, in which BZH at amounts of 1000 mg, 333.3 mg, 200 mg, 142.9 mg, and 111.1 mg was dissolved separately to prepare five solutions for spray-drying (BZH:pullulan = 1:1, 1:3, 1:5, 1:7, and 1:9 *w*/*w*, respectively). A BZH solution (5 mg/mL, 200 mL) was also prepared for spray drying. A laboratory scale spray dryer (Büchi Mini Spray Dryer B-290, Büchi Labortechnik AG, Flawil, Switzerland) was utilized to prepare the spray-dried microparticles for use in an open-cycle system with a pressure nozzle (co-current flow). The processing conditions were as follows: inlet temperature 160 °C, aspiration rate 100%, spray flow rate 600 L/h, and pump setting 25% (6.2 mL/min). These conditions resulted in an outlet temperature of 85 °C. The spray-dried microparticles were recovered from the collection vessel and the spray-dried yields were reported. This process was repeated ten times for each formulation.

### 2.3. Microparticle Characterisation

The mass of freshly prepared microparticles as a function of applied temperature was measured by thermogravimetric analysis (TGA) using the Thermal Advantage TGA Q500 (TA Instrument, New Castle, DE, USA) module. The samples (5–10 mg) were measured in platinum pans and heated at 25–140 °C at a heating rate of 10 °C/min under nitrogen purge. Measurements were made in triplicate.

The morphology of spray-dried microparticles was visualized using scanning electron microscope (SEM, S-570, Hitachi Co., Tokyo, Japan), operated at 15 Kv under high vacuum. Samples of spray-dried microparticles were sputter-coated with a thin layer of gold under partial vacuum (HITACHI E-1010, Tokyo, Japan). Typical micrographs of the spray-dried microparticles were captured as images.

The size distribution of spray-dried microparticles was measured by laser diffraction (Mastersizer 2000, Malvern, UK) with the dry dispersion system. Approximately 200 mg of spray-dried microparticles was used to achieve the required obscuration of 0.5–5%, and the particle size and size distribution were determined. Each sample was measured in triplicate. The average particle size was expressed as the volume-weighted mean. The particle size distribution is expressed in terms of the SPAN factor, which is calculated as: Span = (*d* [*v*, 90] − *d* [*v*, 10])/*d* [*v*, 50], where *d* [*v*, 10], *d* [*v*, 50], and *d* [*v*, 90] are the particle sizes in diameter of a given percentage of particles smaller than the stated size. A higher span value indicates a wider distribution of particle size.

The angle of repose of spray-dried microparticles was determined by a USP 〈1174〉 regulated method (USP, 2016) [36]. For each test, the spray-dried microparticles (~5 g) were poured into a funnel and flowed through a nozzle of approximately 10 mm in diameter down to a platform to generate a conical shape. The angle of repose (θ) was determined by quantifying the height (H) and the diameter (D) of the powder cone, and the angle of repose was calculated by the formula of tgθ = 2H/D.

### 2.4. Analysis of BZH

BZH in the spray-dried powders was quantified by high pressure liquid chromatography (HPLC). Approximately 5 mg of spray-dried powders was dissolved into 1 mL of deionized water, vortexed for 10 min and further sonicated for 30 min to ensure the drug was fully dissolved. Each sample was passed through a cellulose ester syringe filter with pore size of 0.45 µm before HPLC analysis to determine the drug content.

The HPLC instrument of Agilent 1260 infinity consisted of G1312B pump, G1329B autosampler, and G1314F UV detector. For the analysis of BZH, the drug samples (50 µL) were injected into the HPLC column (5 µm C18, 250 × 4.6 mm, 80 Å, ZORBAX Eclipse XDB, Agilent, Santa Clara, CA, USA) and eluted using a mobile phase (flow rate = 0.5 mL/min) of acetate buffer solution (pH3 adjusted by acetic acid): acetonitrile (60:40 *v*/*v*), and detected at 308 nm [37]. The retention time for BZH under these conditions was 6.5 min.

### 2.5. In Vitro Drug-Release Tests

The release of BZH from the spray-dried microparticles was determined by the USP Apparatus II (Paddle Dissolution Apparatus) method as previously reported [20]. Briefly, the RC806 dissolution apparatus (Tianjin University Precision Corporation, Tianjin, China) was employed for the dissolution analysis. For each test, the spray-dried microparticles (200 mg) were dispersed into 900 mL of phosphate buffered saline (PBS, pH 6.8) at 37 °C with stirring at 50 rpm. At scheduled time intervals across 720 min, aliquots of solution (2 mL) were withdrawn from the vessel and filtered through a 0.45 μm cellulose ester filter, and the released drug was quantified by HPLC. A corresponding volume of fresh medium at 37 °C was added into the vessel after each extraction.

### 2.6. Drug-Release Kinetics and Mechanisms

In order to analyze the drug-release kinetics and mechanisms, the drug-release data were fitted into a number of kinetic models including the zero-order model (cumulative mass percentage of drug released versus time, Equation (1)), the first-order model (logarithmic value of cumulative mass percentage of drug remained versus time, Equation (2)), Higuchi’s model (cumulative mass percentage of drug released versus square root of time, Equation (3)), and the Korsmeyer–Peppas model (logarithmic value of cumulative mass percentage of drug released, <60% *w*/*w*, versus logarithmic value of time, Equation (4)) [38].

(1) $$\frac{M_{t}}{M_{\infty }}=K_{0}t$$

(2) $$\frac{M_{t}}{M_{\infty }}=1-e^{-K_{1}t}$$

(3) $$\frac{M_{t}}{M_{\infty }}=K_{H}t^{\frac{1}{2}}$$

(4) $$\frac{M_{t}}{M_{\infty }}=K_{KP}t^{n}$$

where *M_t_* and *M*_∞_ are the amount of drug released at time *t* and after infinite time respectively, and *K*_0_, *K*_1_, and *K_H_* are the release rate constants for zero-order, first-order, and Higuchi diffusion accordingly. *K_KP_* is a constant incorporating structural and geometric characteristics of the dosage form, *n* is the release exponent, which depends on the release mechanism and shape of the matrix tested. Exponent *n* for polymeric controlled delivery systems of spherical geometry has values of *n* ≤ 0.43 for Fickian diffusion, 0.43 < *n* < 0.85 for anomalous (non-Fickian) transport and *n* ≥ 0.85 for Case II (relaxation) transport [39].

### 2.7. Ex Vivo Mucoadhesion Study

The mucoadhesivity of BZH-pullulan spray-dried microparticles was evaluated using a well-defined flow-through method, the principle and conduct of which we have reported previously [20]. This method is designed to assess the mucoadhesive properties of dosage forms administered in the regions of the human body where mucosal tissues are highly affected by the flow of biological fluids. Briefly, the GNP-containing spray-dried microparticles (GNP/pullulan at scheduled mass ratios as described in Section 2.2) were applied to the mucosal membrane of a fresh chicken crop epithelium, to which they adhered by mucoadhesive force, before the mucosal membrane was mounted on a glass slide and inclined at an angle of 45°. A flow of a simulated saliva (pH 6.8, enzyme free), consisting of KCl (0.4 g/L), NaCl (0.4 g/L), CaCl_2_∙2H_2_O (0.906 g/L), NaH_2_PO_4_∙2H_2_O (0.690 g/L), Na_2_S∙9H_2_O (0.005 g/L), and CO(NH_2_)_2_ (1 g/L) [26], was instigated for five minutes to perfuse the surface of mucosal tissue at a flowrate of 0.5 mL/min controlled by peristaltic pump, mimicking the saliva flow rate under normal oral condition. The microparticles with low adhesion force were be washed off and fully dissolved in simulated saliva, and the released GNPs were subsequently determined by UV-Vis (UV-3600, Shimadzu, Japan) at 532 nm [40]. The amount of GNP remaining on the mucosal surface was measured to indicate the proportion of the microparticles retained on the mucosal surface. For investigating the impact of pH values on the mucoadhesivity of pullulan-based microparticles, the simulated saliva was adjusted in pH values to 6.0 and 7.9, respectively, and utilized for testing following the aforementioned process. The mucoadhesion of BZH-only spray-dried microparticles was also investigated as a control. To ensure the reliability and universality of mucoadhesivity data, the chicken crop epithelium was randomly obtained from commercial market and utilized as received, which was outside the regulations on the protection of experimental animals and therefore waived the ethical review and approval.

### 2.8. Statistical Analysis

All data are expressed as means ± standard deviations (S.D.). Statistical analysis was performed by using SPSS one-way ANOVA v24.0 (IBM^®^ SPSS^®^ Statistics) for the comparison of means, and the post-hoc Duncan’s test was employed to state the difference. In all cases, the *p* < 0.05 were considered statistically significant (marked as *p* < 0.05). The terms ‘significant’ or ‘significantly’ mentioned anywhere in this study refer to the parameters that were statistically analyzed by ‘one-way ANOVA, Duncan’s test, *p* < 0.05’.

## 3. Results and Discussions

### 3.1. Particle Characteristics of Spray-Dried Microparticles

The yields of spray-dried microparticles are shown in Table 1. The spray-dried BZH-pullulan microparticles at the mass ratios of 1:0, 1:1, 1:3, 1:5, 1:7, and 1:9 *w*/*w*, labelled as 1R0, 1R1, 1R3, 1R5, 1R7, and 1R9 in sequence, had spray-dried yields in the range of 41.4–46.2% *w*/*w*, with no significant difference observed. The moisture content of spray-dried microparticles was determined by thermogravimetric analysis, and estimated from the weight loss of spray-dried microparticles at temperatures of up to 140 °C. The moisture content was 1.37% *w*/*w* for 1R0 spray-dried microparticles, and significantly higher, 4.5–8.7% *w*/*w*, for all pullulan-modified spray-dried microparticles (Table 1). Additionally, the moisture content was associated with formulations with greater pullulan content: 4.5% *w*/*w* for 1R1, significantly increased to 6.7% *w*/*w* and 6.9% *w*/*w* for 1R3 and 1R5, respectively. In comparison with 1R3, 1R7 and 1R9 had significantly higher moisture contents of 7.8% *w*/*w* and 8.7% *w*/*w*, respectively. The difference in moisture content between 1R9 and 1R5 was also statistically significant. These data were similar to values (6–9% *w*/*w*) previously reported for pullulan-SS spray-dried powders for pulmonary drug delivery [41], and were in the established range for polysaccharide-based spray-dried powders, including 6–7% *w*/*w* for starch [42], 7–9% *w*/*w* for chitosan [20], and 7–10% *w*/*w* for carboxymethylcellulose [43]. The impact of increasing the amount of polysaccharides in formulations on moisture content of spray-dried microparticles can be attributed to the hygroscopicity of polysaccharides which can attract water molecules through hydrogen bonding [41].

**Figure 1:**
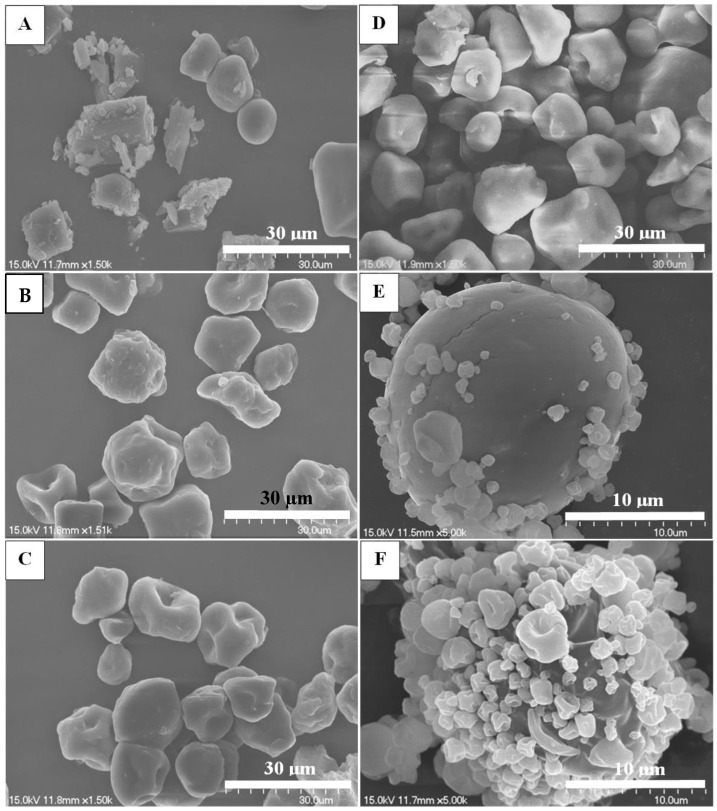
Representative SEM images for the illustration of shape and morphology of BZH-pullulan spray-dried microparticles. (**A**) 1R0; (**B**) 1R1; (**C**) 1R3; (**D**) 1R5; (**E**) 1R7; (**F**) 1R9. Bar = 30 µm for (**A**–**D**) and 10 µm for (**E**,**F**).

Scanning electron microscopy was used to visualize the particle shape, surface morphology, and texture of the BZH-pullulan spray-dried microparticles (Figure 1). It was demonstrated that the primary size was less than 15 μm for 1R0, 1R1, 1R3, and 1R5 (Figure 1A–D). Additionally, the spray-dried 1R0 microparticles exhibited a variety of shapes: spheres, rectangles, and irregular shapes. The rectangle and irregular particles possessed a crystal-like appearance consistent with a report that used X-ray powder diffraction to verify the crystalline state of spray-dried BZH formulations [44]. The spray-dried microparticles of 1R1, 1R3, and 1R5 were sphere-like in shape with crumpled surfaces, which were similar to those images of pullulan-modified spray-dried microparticles reported by Carrige et al. [45]. For formulations of 1R7 and 1R9, with a high mass percentage of pullulan, the majority of spray-dried microparticles were much smaller < 5 μm (Figure 1E,F). These findings were perfectly aligned with our recent findings that pullulan-SS (50:1 *w*/*w*) spray-dried microparticles possessed the size of 4.87 μm (*d* [*v*, 50]) [41], strongly suggesting that the increase in pullulan in the formulation reduces the size of spray-dried microparticles. A few larger spray-dried microparticles (~20 μm) were also observed in the 1R7 and 1R9 formulations, probably due to the influence of BZH, and the reduced sizes made smaller particles have an increased surface energy that caused them be adsorbed to the surface of bigger particles.

**Figure 2:**
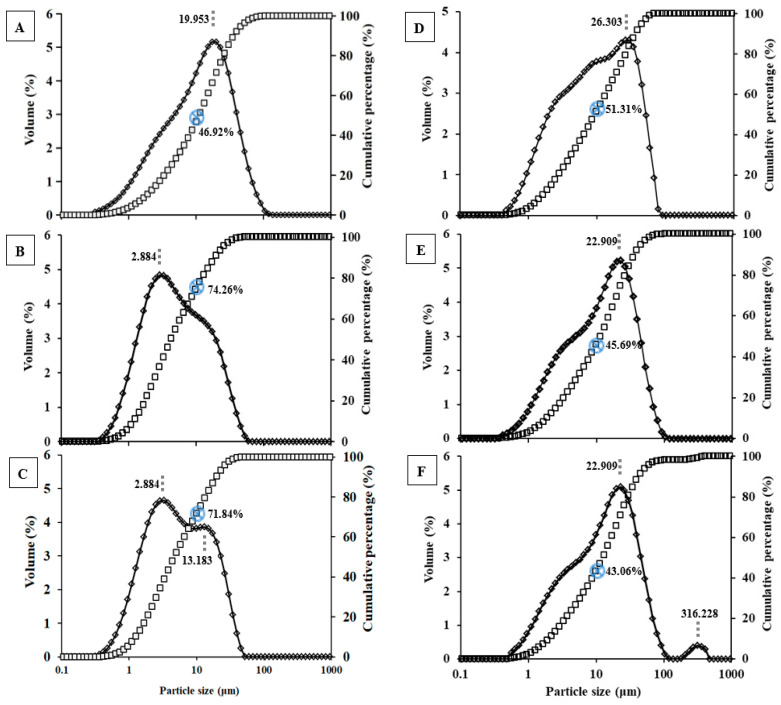
Particle size distribution of BZH-pullulan spray-dried microparticles determined by laser diffraction. (**A**) 1R0; (**B**) 1R1; (**C**) 1R3; (**D**) 1R5; (**E**) 1R7; (**F**) 1R9. (The blue sign of a circle with a cross inside indicates the cumulative percentage in volume (%, *v*/*v*) of microparticles with the size under 10 µm).

To further evaluate the physical size of the spray-dried microparticles, laser diffraction was employed to determine the particle size distribution (PSD, Figure 2). The PSD of each powder was analyzed to indicate the particle interaction and their performance that occurs in the airflow. The PSD of spray-dried 1R0 microparticles demonstrated a broad size distribution of 0.3–105 µm with the mode of 19.95 µm, and the microparticles of <10 µm in diameter occupied a percentage of 46.92% in volume. The spray-dried 1R1 microparticles showed a PSD of 0.3–60 µm with a ‘shoulder’ between 10 and 15 µm, with the mode of 2.88 µm and about 74.26% in volume of <10 µm in diameter. The PSD of spray-dried 1R3 microparticles was bimodal with a major (64% in volume) size of <8 µm, and a second population (36% in volume) with size of 8–53 µm in diameters, for which, the modes were 2.88 µm and 13.18 µm, respectively, and a percentage of 71.84% in volume was less than 10 µm in diameter. For spray-dried 1R5 microparticles, a broad unimodal PSD of 0.5–100 µm was observed with a ‘shoulder’ existing at ~3–13 µm, the mode was 26.30 µm, and the powders of <10 µm in diameter occupied 51.31% in volume (Figure 2D). In comparison with spray-dried 1R7 microparticles with the unimodal PSD of < 100 µm (mode: 22.91 µm; 45.69% in volume <10 µm), the 1R9 spray-dried microparticles displayed bimodal PSD with a major population of <100 µm (98.1% in volume; mode: 22.91 µm) and a second PSD of 158–478 µm (1.9% in volume; mode: 316.23 µm), and had a reduced percentage to 43.06% in volume (vs. 45.69% for IR7) for microparticles of <10 µm. These data suggested that the drug BZH, at the mass ratios of 1:1 w/w and 1:3 w/w to pullulan in the formulation, can create spray-dried powders with good dispersibility in the airflow. With a further increase in pullulan content in the formulations, the spray-dried microparticles tended to be aggregated, probably due to the greater moisture content generated. For better understanding the spray-dried microparticles of BZH-pullulan at different mass ratios, the mean diameter of D [4,3] and the SPAN are reported (Table 1). The D [4,3] of spray-dried BZH-pullulan microparticles was 18.59 µm for 1R0 (BZH-only) and markedly decreased to 9.02 µm for 1R1 (50% pullulan), confirming the addition of pullulan can effectively reduce the mean size. With a further increase in pullulan content in the formulation, the mean sizes of spray-dried microparticles were incrementally enlarged to 10.49 µm for 1R3, 15.53 µm for 1R5, 18.80 µm for 1R7, and 24.69 µm for 1R9, presumably as aggregation of the spray-dried microparticles increased. The reasons may be that the increases in pullulan content in the formulations were associated with higher moisture content and reduced geometric dimensions (i.e., for 1R7 and IR9) that led to increased surface energy and powder agglomerates. Further, for all spray-dried microparticles, the span was over 3, suggesting a broad size distribution. These data aligned well with the observations of PSD.

The angle of repose characterizes the flowability of dry powders, and is analyzed following the methods specified in the US and European Pharmacopoeia. The angle of repose is the angle formed between the side of a stationary pile of powder and the horizontal. The greater angle of repose suggests a stronger interparticle cohesion. Although there is some variation in the qualitative description of powder flow using the angle of repose, much of the pharmaceutical literature appears to be consistent with the classification by Carr [46], where powders with angle of repose in the range of 25–45° are generally considered to have satisfactory flowability for pharmaceutical processing. This has been codified by USP and listed in chapter <1174> [36]. The angle of repose was 25.8° for BZH-only dry powder and significantly greater at 30.3–36.5° for all pullulan-based spray-dried microparticles (Table 1). Despite the impact of pullulan, all of these spray-dried microparticles had an angle of repose < 45°, indicating good flowability. These data were consistent with findings for spray-dried microparticles consisting of pullulan and SS (50:1 *w*/*w*), which had the angle of repose of 30–36° [41]. Interestingly, the angles of repose for 1R5–1R9 spray-dried microparticles did not show significant differences despite their moisture contents increasing from 6.9% *w*/*w* to 8.7% *w*/*w*.

### 3.2. In Vitro Drug-Release Study

**Figure 3:**
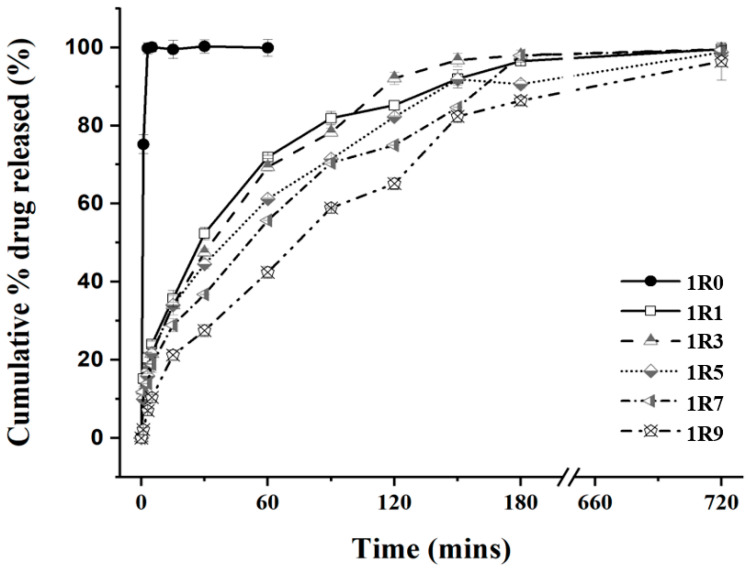
In vitro sustained drug-release profiles of spray-dried microparticles. The USP Apparatus II (Paddle Dissolution Apparatus) method was employed for the examination of BZH release from the spray-dried microparticles. For each test, the spray-dried microparticles at a certain amount (200 mg) were added into 900 mL of PBS (37 °C, 50 rpm). At scheduled time intervals, aliquots of solution (2 mL) were withdrawn (the same quantity of PBS refilled accordingly) from the vessel for the analysis of released drug by HPLC. The cumulative amount of mass in percentage was plotted as a function of the time for release.

The calibration curve of BZH was expressed as Y = 36.269 X − 26.306 (R^2^ = 0.9993), where Y and X were the UV absorbance and drug concentration, respectively. Figure 3 shows the release profiles of BZH from the BZH-pullulan spray-dried formulations, and the BZH-only spray-dried powder that was used as the control. Rapid drug dissolution was observed from the BZH-only (1R0) spray-dried microparticles, with 100% *w*/*w* of drug dissolved into the dissolution medium within 2 min. In contrast, the addition of the pullulan into the formulation effectively lengthened the time of drug release, and all BZH-pullulan spray-dried microparticle formulations demonstrated sustained drug-release profiles over 180 min. The increase in pullulan content in the formulation generally reduced the rate of drug release. The spray-dried microparticles of 1R1, 1R3, 1R5, 1R7, and 1R9 released drug at cumulative mass percentages of 71.9%, 69.4%, 61.2%, 55.7%, and 42.4% *w*/*w* over 60 min, of 85.2%, 92.9%, 82.2%, 75.0%, and 65.1% w/w over 120 min, and 97.0%, 96.5%, 91.9%, 90.6%, and 86.4% *w*/*w* over 180 min, respectively, which clearly demonstrated that the liberation of drug molecules from the matrix was dependent on the pullulan-content. All formulations released >80% of the drug in 180 min and 95–98% *w*/*w* of the drug in 12 h, showing that BZH molecules disassociation from pullulan polymer chains in a way that will enable therapeutic effects.

To interrogate drug-release kinetics and mechanisms from spray-dried pullulan-based microparticles, the release data were fitted into the zero-order model, the first-order model, Higuchi’s model, and the Korsmeyer–Peppas model (Equations (1)–(4)). With the zero-order model, the rate of drug release is constant and independent of the drug concentration or amount of drug remaining in the delivery system; for example, when the relaxation of polymer chains as a result of glass transition to rubbery state upon water uptake is the decisive factor for the rate of drug release [47]. With the first-order model, the rate of drug release is controlled by the difference between drug concentrations inside the delivery system compared to the surrounding medium, and it is further related to the rate of water permeation and the rate of polymer gelation [48]. The Higuchi model describes the rate of drug release from a planar surface or a sphere. The model is built upon Fickian diffusion and is applicable when the rate of drug release is reliant on molecule diffusion through the matrix [49]. With the Korsmeyer–Peppas model, the first 60% of drug-release data are fitted into the equation to identify the mechanism of drug release [38]. For spheres, a value of n ≤ 0.43 indicates drug release controlled by Fickian diffusion, values 0.43 < n < 0.85 designate a mechanism of anomalous or non-Fickian transport where the release of drug molecules is jointly controlled by drug diffusion and polymer erosion as occurs with the process of polymer swelling, chain relaxation, and disentanglement [50], and values of n ≥ 0.85 suggest a mechanism of case-II transport which is associated with polymer relaxation or degradation [51,52].

When the release data for all spray-dried BZH-pullulan microparticles were fitted into each of above-mentioned kinetic models, the correlation coefficient (R^2^) values were <0.95 for fitting into the equation of the zero-order model, but >0.98 for equations associated with all other models (Table 2). This suggests that the rate of drug release was determined by the gradient of drug concentration inside microparticles against surrounding solution and cooperatively controlled by Fickian diffusion and polymer chain relaxation [53]. For spray-dried microparticles with decreasing BZH content from 1R1 to 1R9, the overall release rate constant was reduced correspondingly, from 0.016 min^−1^ down to 0.010 min^−1^ in the first-order model, from 6.85 min^−1/2^ to 6.51 min^−1/2^ in the Higuchi model, and from 14.22 min^−n^ to 2.80 min^−n^ in the Korsmeyer–Peppas model. The t_50_, time required for release 50% *w*/*w* of BZH in each powder, concomitantly increased from 30.8 min to 65.5 min and from 32.7 min to 67.8 min for the first-order and Higuchi models, respectively. These data support the interpretation that the rate of drug release was reliant on drug concentration remaining in the device as the BZH content was 50% *w*/*w* in 1R1 spray-dried microparticles, and then systematically reduced in following formulations to 10% *w*/*w* in 1R9.

The percentage of drug released at time 0 (P_0_) was also estimated based on linear regression in the Higuchi model. The P_0_ was 10.8%, 6.3%, 6.0%, and 3.6% *w*/*w* for the spray-dried microparticles of 1R1, 1R3, 1R5, and 1R7, respectively, and zero release for 1R9. This can be explained by the reducing drug content in formulations resulting in fewer drug molecules distributed on the surface of spray-dried microparticles, hence the lower initial drug concentration upon instantaneous dissolution once contact is made with the medium. By extension, for the 1R9 spray-dried microparticles, the absence of an initial burst of drug release indicated that there were no BZH molecules distributed on the particle surfaces and available for instantaneous dissolution in the medium for detection at t_0_. Consistent with this, there was no lag time on releasing drug from spray-dried microparticles of 1R1–1R7, but a lag time of 47.5 s was required for 1R9 spray-dried microparticles to allow water uptake, channel opening, and release of drug molecules via diffusion.

The exponent of n in the Korsmeyer–Peppas model is an indicator of the mechanism for drug release. The spray-dried microparticles of 1R1–1R7 showed a value of n in the range of 0.36–0.43, which suggests that Fickian diffusion is the mechanism that governs drug release. However, the analysis of release profile for spray-dried 1R9 microparticles found the value of n to be 0.69, indicating an anomalous (i.e., non-Fickian) transport mechanism where the drug-release rate is jointly controlled by drug diffusion and polymer erosion. These findings can be contextualized by reference to similar studies. In a study described by Soni and Ghosh [54], the drug release from pullulan-modified microparticles was also modelled the best by first-order kinetics, indicating a concentration-dependent rate of drug release. Moreover, the value of R^2^ was >0.98 for Higuchi model, suggesting drug release was largely controlled by Fickian diffusion. For the Korsmeyer–Peppas model, the value of n was in the range of 0.37–0.63 (for over half the formulations n was ≤0.43), signifying that the rate of drug release from pullulan was primarily controlled by Fickian diffusion, together with polymer erosion in those formulations that incorporated supplementary compositions. In another study, the pullulan-based microparticles were employed to control the release of naproxen, and the value of n was verified as <0.43 for all situations, further confirming the Fickian diffusion as the primary release mechanism [55]. In 2017, we reported the use of chitosan as a matrix to create spherical drug-delivery systems, where the release of BZH can be extended to ~20 min. In this study, the use of pullulan as a matrix markedly lengthened the release time (9× longer) for BZH from spray-dried microparticles. In future, it may be possible to combine chitosan with pullulan to fine tune the rate of drug release and more precisely adjust the drug-release time to satisfy specific clinical needs.

### 3.3. Ex Vivo Mucoadhesion Study

The ex-vivo mucoadhesive features of spray-dried microparticles were estimated using a method developed for testing mucoadhesivity of spray-dried microparticles under conditions of fluid perfusion [20]. The chicken crop epithelium is considered as an appropriate model mucosa for the assessment of mucoadhesion interaction, as it produced data that were consistent with clinic observations in which the dry powders were directly applied to human buccal mucosa [56]. The influence of drug dissolution during perfusion flux was eliminated by using an equivalent amount of insoluble GNPs to replace BZH in each formulation of spray-dried microparticles in this study. The extent of GNP retention was used as an index of mucoadhesion of the spray-dried microparticles to the mucosal membrane. The retention rates for all formulations of 1R1–1R9 are listed in Table 3, with data presented for perfusate at three different pH values. As previously reported, only 3.3–3.6% *w*/*w* of unencapsulated GNP remained on the mucosa after the being perfused by buffer, suggesting poor mucoadhesion ability associated with GNP themselves. When encapsulated in pullulan, the retention of formulation on the mucosa was markedly enhanced. For the 1R1 spray-dried microparticle (50% *w*/*w* of pullulan), GNP retention on mucosa was significantly increased (compared to 1R0) to 37.0% *w*/*w* (pH 6.0), 43.4% *w*/*w* (pH 6.8), and 35.1% *w*/*w* (pH 7.9), suggesting pullulan can greatly improve the mucoadhesion of microparticles under relevant pH conditions. For 1R3 spray-dried microparticle (75% *w*/*w* of pullulan), GNP retention on the mucosa was incrementally greater with 40.6% *w*/*w* (pH 6.0), 46.3% *w*/*w* (pH 6.8), and 39.8% *w*/*w* (pH 7.9), which was attributed to the increased pullulan content in formulation. Indeed, as the content of pullulan in spray-dried microparticles was increased, the GNP retention rate on the mucosa continued to be augmented, to 59.2–66.4% *w*/*w* for 1R5, 68.1–69.7% *w*/*w* for 1R7, and 70.1–78.8% *w*/*w* for 1R9. Retention by the mucosa was significantly greater for the spray-dried microparticles with pullulan mass percentage over 83.3% *w*/*w* (1R5–1R9) compared to the retention of 1R1 and 1R3 spray-dried microparticles. For all formulations, there was no effect on retention of varying the perfusate between pH 6 and pH 7.9. This was expected as the viscosity of pullulan is reported to be a key factor to determine mucoadhesivity [57] and is ‘essentially unaffected by pH over a wide range of pH values (from <2 to >11)’ [58].

The adhesion mechanism of pullulan onto mucosal membrane is through contact and consolidation and can be supported by a combination of different theories [59,60,61]. At the contact stage, the hydrophilic pullulan-based spray-dried microparticles were applied to the mucosa and were wetted by aqueous fluid. According to wetting theory, the high surface area and energy of microparticles may augment the spreading of liquid and enhance the work of adhesion upon contact. In the consolidation step, the penetrated water can act as a plasticizer to swell the matrix and relax pullulan chains to enable them to interact with biomacromolecules of glycoprotein (i.e., mucin) in the mucus by van der Waals and hydrogen bonds, leading to entangled complexes that enhance mucoadhesion. This mechanism has been observed using atomic force microscopy to study the complexes formed by a biopolymer of pectin with mucus glycoproteins through entanglement and secondary bonds that enhance mucoadhesion [62].

## 4. Conclusions

This study produced pullulan-based spray-dried microparticles with a mean size less than 20 µm and powder flowability properties that are indicative of processability. The inclusion of pullulan in the delivery system provides a matrix that can control drug release and effectively extend the release time over 180 min, nine times longer than that of a previously studied chitosan-based particulate delivery system. Analysis of drug-release data using different models found that the drug-release kinetics were indicative of concentration dependence, and jointly controlled by drug diffusion and polymer erosion. The inclusion of pullulan also induced microparticles to adhere to the mucosa membrane regardless across a biorelevant range of pH. These data clearly demonstrate the potential of BZH-pullulan spray-dried microparticles to be further developed as drug-delivery systems for more effective local treatment of oral mucosal diseases.

## Figures and Tables

**Table 1:** Physical characteristics of pullulan-based spray-dried microparticles. The data were expressed as the mean ± S.D. (n = 10 for spray-dried yield, and n = 3 for all others).

SDM	Yield (% *w*/*w*)	Moisture Content (% *w*/*w*)	Repose Angle (^o^)	Particle Size Distribution				
				*d* [*v*,10] (μm)	*d* [*v*,50] (μm)	*d* [*v*,90] (μm)	Span	D [4,3] (μm)
1R0	43.5 ± 3.8 ^I^	1.37 ± 0.44 ^I^	25.8 ± 0.9 ^I^	1.84	11.01	36.08	3.11	18.59
1R1	41.6 ± 4.8 ^I^	4.5 ± 0.3 ^II^	30.3 ± 1.1 ^II^	1.21	4.32	19.23	4.18	9.02
1R3	46.2 ± 3.1 ^I^	6.7 ± 0.1 ^III^	33.3 ± 1.7 ^III^	1.24	4.66	19.60	3.94	10.49
1R5	46.1 ± 5.1 ^I^	6.9 ± 0.5 ^III, IV^	35.3 ± 1.6 ^III, IV^	1.67	9.62	37.61	3.74	15.35
1R7	46.2 ± 4.2 ^I^	7.8 ± 0.8 ^IV, V^	35.7 ± 1.2 ^III, IV^	1.91	11.55	37.02	3.04	18.80
1R9	41.4 ± 5.9 ^I^	8.7 ± 0.7 ^V^	36.5 ± 1.8 ^IV^	2.01	12.68	42.87	3.22	24.69

SDM: Spray-dried microparticles. The data in the same column labelled with the same Roman numeral (i.e., I, II, III, IV, V) as a subscript indicates no significant difference.

**Table 2:** Analysis of drug-release kinetics and mechanisms by different models.

SDM	Zero-Order		First-Order			Higuchi					Korsmeyer–Peppas			
	R^2^	K_0_ (min^−1^)	R^2^	K_1_ (min^−1^)	t_1,50_ (min)	R^2^	K_H_ (min^−1/2^)	P_0_ (%)	t_L_ (s)	t_H,50_ (min)	R^2^	K_KP_ (min^-n^)	n	M
1R1	0.8833	0.45	0.9876	0.016	30.8	0.9798	6.85	10.8	N/A	32.7	0.9811	14.22	0.36	F
1R3	0.9088	0.49	0.9846	0.021	30.9	0.9884	7.41	6.3	N/A	34.8	0.9957	11.73	0.40	F
1R5	0.9152	0.45	0.9755	0.013	40.2	0.9912	6.80	6.0	N/A	43.7	0.9960	10.36	0.43	F
1R7	0.9499	0.45	0.9853	0.012	47.8	0.9961	6.63	3.6	N/A	49.0	0.9734	9.55	0.39	F
1R9	0.9120	0.46	0.9786	0.010	65.2	0.9915	6.51	N/A	47.5	67.8	0.9763	2.80	0.69	A

SDM—spray-dried microparticles; R^2^—correlation coefficient; K_0_, K_1_, K_H_, K_KP_—release rate constant of the zero-order model, first-order model, Higuchi model, and Korsmeyer–Peppas model, respectively; t_1,50_, t_H,50_—times required to release 50% *w*/*w* of BZH, calculated from the first-order and Higochi models, correspondingly; t_L_—lag time; n—exponent in Korsmeyer–Peppas model; M—mechanism for drug release; F—Fickian diffusion; A—anomalous transport.

**Table 3:** The percentage of spray-dried microparticles retained on the mucosa. (all data presented as mean ± SD, n = 3).

pH	Spray-Dried Microparticles					
	1R0	1R1	1R3	1R5	1R7	1R9
6.0	3.5 ± 0.3 ^a, I^	37.0 ± 5.3 ^b, I^	40.6 ± 5.3 ^b, I^	59.2 ± 6.4 ^c, I^	69.7 ± 8.7 ^c, I^	70.1 ± 6.2 ^c, I^
6.8	3.6 ± 0.4 ^a, I^	43.4 ± 7.9 ^b, I^	46.3 ± 7.2 ^b, I^	66.4 ± 5.2 ^c, I^	68.8 ± 9.6 ^c, I^	78.8 ± 8.4 ^c, I^
7.9	3.3 ± 0.3 ^a, I^	35.1 ± 3.9 ^b, I^	39.8 ± 9.6 ^b, I^	65.2 ± 5.7 ^c, I^	68.1 ± 6.6 ^c, I^	74.5 ± 5.4 ^c, I^

The same Roman numeral (i.e., Ⅰ) and letter (i.e., a, b or c) as a subscript indicate no significant difference for those data in the same column and row respectively.

## Data Availability

The data can be shared upon request.
